# Evaluation of the implementation of clinical simulation as a learning tool: an 18-month experience at a university hospital

**DOI:** 10.3389/fmed.2026.1770488

**Published:** 2026-05-04

**Authors:** Munir Mohamed Mimun, Telmo Raul Aveiro-Róbalo, Esther Jovell-Fernández, Ester Cañadell Yetano

**Affiliations:** 1 Department of Internal Medicine, Consorci Sanitari de Terrassa, Hospital Universitari, Terrassa, Spain; 2 Family and Community Medicine, Consorci Sanitari de Terrassa, Hospital Universitari, Terrassa, Spain; 3 Department of Epidemiology, Consorci Sanitari de Terrassa, Hospital Universitari, Terrassa, Spain; 4 Department of Medicine, School of Medicine and Health Sciences, Universitat Internacional de Catalunya, Barcelona, Spain; 5 Department of Paediatry, Consorci Sanitari de Terrassa, Hospital Universitari, Terrassa, Spain; 6 Simulation Committee, Consorci Sanitari de Terrassa, Hospital Universitari, Terrassa, Spain

**Keywords:** clinical simulation, cross sectional analysis, kirkpatrick and kirkpatrick learning model, medical simulation, multidisciplinary team, simulation nursing education

## Abstract

**Introduction:**

Clinical simulation has become established as a key tool in healthcare education; however, evidence regarding its multidisciplinary implementation in university hospitals, integrating distinct professional profiles into institutional programs, remains limited. The objective of this study was to evaluate the satisfaction and perceived impact of clinical simulation following its structured implementation over 18 months in a university hospital.

**Materials and methods:**

An observational, descriptive, cross-sectional study was conducted, corresponding to Level 1 of the Kirkpatrick model. The study included attending physicians, medical residents, nursing staff, and nursing assistants from the *Hospital Universitari Consorci Sanitari de Terrassa* (Catalonia, Spain) who participated in simulation activities between March 2024 and August 2025. Satisfaction was assessed using the validated Spanish version of the Clinical Simulation Quality and Satisfaction Survey by Durá Ros (14 items, 1–5 Likert scale). A descriptive analysis by professional category and a correlation analysis between age and global satisfaction were performed. The statistical significance level considered for the analysis was set at 5%.

**Results:**

A total of 542 valid questionnaires were analyzed (mean age 37 ± 11 years; 76.9% women). Although all dimensions showed mean scores above 4.5/5, comparative analysis revealed statistically significant differences according to professional profile. The nursing group achieved the highest scores, particularly in activity prioritization (4.97 ± 0.17; *p* < 0.001) and communication (*p* = 0.028). Specifically, for overall satisfaction (*p* = 0.003) and perceived improvement in competencies (*p* = 0.042), *post hoc* analysis confirmed significant differences between the nursing group and the TCAE/Other group (adjusted *p* = 0.030 and adjusted *p* = 0.036, respectively). No significant correlation was found between age and overall satisfaction (*ρ* = −0.027; *p* = 0.52).

**Conclusion:**

The structured implementation of clinical simulation in a university hospital was associated with very high levels of acceptance among professionals from different categories, even in the absence of a dedicated simulation center. The multidisciplinary approach of this study—integrating attending physicians, residents, nurses, and nursing assistants—suggests the transversal utility of simulation in real healthcare teams within hospital continuing education. However, given the cross-sectional design and the focus on Level 1 outcomes, further research using longitudinal or controlled methodologies is required to confirm these findings and to objectively evaluate skill acquisition, transfer to clinical practice, and long-term healthcare impact.

## Introduction

Healthcare education has progressively incorporated methodologies aimed at improving patient safety and optimizing the acquisition of technical and non-technical skills. Clinical simulation has become established as an essential strategy in training programs, supported by experiential learning models that allow for the safe training of skills without risk to patient care (1, 2). As health systems face increasing complexity, simulation has evolved from a complementary tool into a pillar of continuing professional development (3), aligning with educational theories that favor the improvement of clinical decision-making and technical skills (4).

Various meta-analyses indicate that repetitive practice in simulated environments favors acquisition and retention of knowledge superior to traditional teaching techniques (5–7). Furthermore, the impact of these interventions extends specifically to non-technical skills, demonstrating tangible improvements in clinical reasoning, decision-making, and teamwork in crisis situations (8).

Internationally, simulation has generated robust data. Experiences such as those in Saudi Arabia and the United States have reported significant improvement in clinical self-efficacy (9) and an objective reduction in clinical errors through cognitive aids (10). Studies in China have evidenced the technical superiority of high-fidelity simulation compared to computer-based methods (11). In the Philippines, high levels of satisfaction have been validated even with low-cost simulators (12). In the European context, the situation presents greater heterogeneity. Despite the existence of innovative collaborations, such as in obstetric training (13), implementation is not uniform. National surveys in Italy reveal that a majority of residents perceive their simulation training as insufficient due to the lack of structured programs (14).

In Spain, recent evidence suggests a positive trend in the consolidation of this methodology. Research in primary care has documented notable increases in knowledge following training (15), and studies in the north and south of the country report high satisfaction indices related to the realism of cases and theory-practice integration (16, 17). However, the literature continues to show relevant gaps regarding systematic application in general hospitals and its impact on active professionals (1, 18). Given the decentralized organization across different autonomous communities of the Spanish healthcare system and existing structural challenges (19, 20), it is a priority to have studies that analyze the comprehensive institutional experience during the consolidation phase of these programs, providing scientific evidence under a multidisciplinary approach to clinical simulation.

In this context, the present study evaluates the structured implementation of clinical simulation in a university hospital over a period of 18 months. The objective is to analyze satisfaction levels and the perceived impact by different professional groups, providing evidence on the optimization of professional development strategies in the European hospital environment.

## Materials and methods

### Study design

An observational, descriptive, and cross-sectional study was conducted. The research is framed within Level 1 of the Kirkpatrick model, focusing on satisfaction and perception of learning following simulation-based training activities.

### Setting and participants

The study was carried out at the Hospital Universitari Consorci Sanitari de Terrassa (CST), in Catalonia, Spain. Professionals from different categories were included (attending physicians, medical residents, nursing staff, and nursing assistants) who participated in clinical simulation activities developed within the framework of CST institutional training programs between March 2024 and August 2025. Non-probability convenience sampling was used. For the sample size calculation, an expected frequency of 50%, a 95% confidence interval, and a 5% margin of error were considered. Based on this, the minimum sample size for an infinite population was calculated at 385. In total, 542 valid questionnaires from 45 simulation activities were analyzed.

### Intervention procedure

Clinical simulation activities were carried out in adapted spaces within the hospital, as the institution does not possess a dedicated simulation center. Workgroups consisted of 10–15 participants. Each session began with an introduction of approximately 20 min aimed at generating a safe environment and explaining the activity’s objectives. Subsequently, participants performed two or three simulated clinical scenarios, each lasting 10–20 min. Following each scenario—which were medium-fidelity simulations—a structured debriefing of 20 to 30 min was conducted by a facilitator, promoting reflection on knowledge, skills, and attitudes. The average duration of each session was 3 hours.

### Measurement instrument

The validated Spanish version of the Clinical Simulation Quality and Satisfaction Survey by Durá Ros (19) was used, designed to evaluate learner perception regarding simulation as a learning tool. The instrument consists of 14 items scored on a 5-point Likert scale (1: “strongly disagree” to 5: “strongly agree”).

For the analysis of results, the arithmetic mean of the item scores was calculated; each item evaluates a specific aspect of the clinical simulation experience, including a final item that captures global satisfaction with the activity. Interpretation of results was performed using the instrument’s validation criteria: values greater than 4 indicate high satisfaction, scores between 3 and 4 reflect a moderate or neutral perception, and values lower than 3 indicate areas of dissatisfaction or those susceptible to improvement (19).

The dimensions evaluated include teaching utility, scenario realism, technical and non-technical skills, theory–practice integration, instructor training, and global satisfaction. The survey was administered in digital format via Microsoft Forms (Office 365) through a QR code provided at the end of each activity, guaranteeing anonymity in the collection of quantitative and qualitative data.

### Variables

Sociodemographic variables (age, gender, professional category, and year of residency for trainee doctors) and variables related to satisfaction were collected, evaluated via the 14 questionnaire items. Each item was analyzed individually. Global satisfaction was specifically evaluated through the item “Overall, the clinical simulation experience has been satisfactory,” in accordance with the design and original validation of the instrument.

### Statistical analysis

Data were exported to Microsoft Excel and analyzed using SPSS v29.0 (IBM Corp., Armonk, NY, USA). A descriptive analysis of the variables was performed: qualitative variables were expressed as absolute frequencies and percentages, and quantitative variables as means and standard deviations or medians and interquartile ranges, depending on their distribution. To compare satisfaction scores between professional categories, the analysis of variance (ANOVA) test was applied; if application conditions were not met, the Kruskal–Wallis test was used. The Bonferroni correction was applied for multiple comparisons to identify between-group differences when statistically significant results were observed. The correlation between age and global satisfaction, evaluated via an ordinal item on the questionnaire, was analyzed using Spearman’s correlation coefficient (*ρ*). A value of *p* < 0.05 was considered significant.

### Confidentiality, data protection, and ethical declaration

This study adhered to bioethical standards for research involving human subjects established in the Declaration of Helsinki. Data were handled confidentially using coded identifiers, and the study protocol was approved by the Clinical Research Ethics Committee of the Consorci Sanitari de Terrassa. Compliance with Spanish Law 14/2007 on Biomedical Research and Regulation (EU) 2016/679 on data protection was ensured. The study approval code (CEIm) was 02-24-308-009.

## Results

### Participant characteristics

During the period between March 2024 and August 2025, 542 valid questionnaires were collected corresponding to clinical simulation activities performed at the Hospital Universitari Consorci Sanitari de Terrassa (CST). The mean age of participants was 37 ± 11 years. Women accounted for 76.9% (417) of the sample.

Regarding professional category, the largest group was nursing staff, with 200 participants (37.0%), followed by resident physicians (MIR) with 115 (21.3%), attending physicians with 45 (8.3%), and nursing assistants and other healthcare staff with 181 participants (33.5%). Table 1 shows the percentage distribution of professional categories, after grouping minority profiles under the label “Others” (which includes 20 radiologic technicians, 17 pharmacy technicians, 10 administrative personnel, 20 physical therapists, and 3 emergency medical technicians).

**Table 1:** Sociodemographic characteristics of the study sample.

Characteristic gender	Frequency (*n*)	Percentage (%) or mean (SD)
Female	417	76.9
Male	124	22.9
Non-binary	1	0.2
Mean age	542	37 ± 11
Professional category		
Nurses	200	37
Nursing Assistants and Other Healthcare Staff (NA/Others)	181	33.5
Medical Internal Residents (MIR)	115	21.3
Attending Physicians (Attending)	45	8.3
System lost	1	0.2
Total	542	100.0

### Satisfaction results from the clinical simulation quality and satisfaction survey (Durá Ros)

Statistically significant differences were observed in the distribution of scores between groups across several dimensions. Regarding the prioritization of clinical activities (*p* < 0.001), the nursing group recorded the highest mean score (4.97 ± 0.17).

Regarding global satisfaction, significant overall differences were found (*p* = 0.003). Detailed post-hoc analysis revealed that these differences were statistically significant between Nursing and the Nursing assistants/Others group (adjusted *p* = 0.030), although the comparison between Residents and Nursing did not reach statistical significance after adjustment (adjusted *p* = 0.06).

In the communication dimension (global *p* = 0.028), nursing staff awarded the highest ratings (4.97 ± 0.17). Differences were more marked between Nursing and Nursing assistants/Others (adjusted *p* = 0.057), and between Residents and Nursing (unadjusted *p* = 0.030), although the latter lost statistical significance after adjustment.

Regarding improvement in clinical competence (*p* = 0.042), significant differences were specifically located between the Nursing assistants/Others group and Nursing (adjusted *p* = 0.036).

Finally, in the dimensions of clinical reasoning (*p* = 0.048) and Teaching Utility (global *p* = 0.064), although overall differences were tighter, the analysis suggested higher scores in Nursing compared to Nursing assistants/Others; while not reaching statistical significance (Table 2).

**Table 2:** Mean scores of the satisfaction survey items grouped by professional category (1–5 Likert scale).

Satisfaction survey items	Attending physicians (mean [SD])	MIR (Mean [SD])	Nurses [Mean (SD)]	NA/others (Mean [SD])	*p*-value	Effect size (*n*^2^*p*)
Teaching utility	4.89 (0.38)	4.95 (0.22)	4.98 (0.16)	4.90 (0.35)	0.064	0.039
Scenario realism	4.58 (0.58)	4.62 (0.59)	4.58 (0.67)	4.63 (0.60)	0.867	0.007
Technical skills	4.87 (0.34)	4.80 (0.42)	4.79 (0.50)	4.79 (0.48)	0.843	0.010
Clinical reasoning	4.93 (0.33)	4.92 (0.27)	4.93 (0.27)	4.85 (0.41)	**0.048**	0.035
Adaptation to theoretical knowledge	4.80 (0.55)	4.87 (0.34)	4.85 (0.38)	4.81 (0.47)	0.805	0.016
Safety–confidence	4.64 (0.64)	4.72 (0.50)	4.78 (0.48)	4.70 (0.56)	0.401	0.021
Theory–practice integration	4.76 (0.53)	4.83 (0.42)	4.90 (0.30)	4.79 (0.47)	0.065	0.039
Motivation	4.84 (0.42)	4.89 (0.32)	4.94 (0.25)	4.83 (0.43)	0.056	0.040
Case duration	4.71 (0.51)	4.72 (0.57)	4.66 (0.65)	4.66 (0.66)	0.887	0.010
Instructor competence	4.96 (0.21)	4.96 (0.20)	4.97 (0.17)	4.93 (0.26)	0.293	0.022
Communication	4.89 (0.32)	4.89 (0.37)	4.97 (0.17)	4.89 (0.35)	**0.028**	0.040
Prioritization of clinical activities	4.82 (0.39)	4.87 (0.34)	4.95 (0.25)	4.78 (0.51)	**<0.001**	0.061
Improvement in clinical competence	4.87 (0.40)	4.83 (0.40)	4.89 (0.33)	4.77 (0.48)	**0.042**	0.039
Global satisfaction	4.89 (0.32)	4.86 (0.35)	4.97 (0.17)	4.88 (0.36)	**0.003**	0.052

Values are presented as mean (standard deviation). *p*-values derived from ANOVA or Kruskal–Wallis tests as appropriate. Effect sizes are reported as partial eta squared (*η*^2^*p*). Bold values indicate statistical significance (*p* < 0.05).

In addition to statistical significance testing, effect sizes were calculated for all comparisons using partial eta squared (*η*^2^*p*). According to conventional benchmarks (small ≥ 0.01, moderate ≥ 0.06, large ≥ 0.14), statistically significant differences were generally associated with small effect sizes, including communication (*η*^2^*p* = 0.040), improvement in clinical competence (*η*^2^*p* = 0.039), clinical reasoning (*η*^2^*p* = 0.035), and global satisfaction (*η*^2^*p* = 0.052). The dimension “prioritization of clinical activities” showed a moderate effect size (*η*^2^*p* = 0.061).

### Relationship between age and satisfaction

The relationship between age and the global satisfaction item was analyzed. The correlation was very low and inversely proportional (*ρ* = −0.027; *p* = 0.52), without statistical significance.

## Discussion

The results of this study show very high satisfaction levels among participating professionals, with global scores exceeding 4.5/5 in all evaluated dimensions. This main finding aligns with international literature, which describes clinical simulation as an effective and well-valued methodology for the acquisition of technical and non-technical skills, reinforcing its role as a central training tool in complex healthcare environments (1–3).

The data obtained evidence a high global satisfaction, suggesting that this positive perception is a transcultural phenomenon. When contrasting these results with international literature, similarities are observed with interprofessional simulation programs in Taiwan, where satisfaction scores reached 4.42/5 in hospital nursing teams (21), and with experiences in the Philippines, where satisfaction with low-cost models exceeded 90% (12), confirming the trend observed in the analyzed sample.

Nevertheless, it should be considered that high satisfaction scores in simulation-based education frequently present a ceiling effect, particularly when Likert scales are used and participation is voluntary. The clustering of responses near the upper limit of the scale in our sample suggests a potential restriction of variance, which may reduce the instrument’s discriminatory capacity between professional groups in high-satisfaction contexts. Therefore, although the results are clearly positive, caution is required when interpreting satisfaction as a surrogate marker of educational effectiveness, as it does not necessarily translate into measurable improvements in clinical performance or patient outcomes.

However, the high satisfaction obtained in the present sample contrasts significantly with the reality observed in other nearby European environments. The fact that the study sample shows such a positive valuation could suggest that the program structure and instructor training have managed to overcome implementation barriers, unlike what has been reported in Italy, where 73% of residents rated their simulation training as “less than good” (14).

At the national level, the data reinforce the positive trend toward clinical simulation; for instance, the high valuation of the theory-practice relationship in this work is consistent with findings in Cantabria, where 87% of participants reported high satisfaction highlighting the realism of cases (16). Similarly, the subjective perception of competence improvement recorded in the present study complements findings from systematic reviews documenting improvements in self-confidence, technical skills, and the theory-practice relationship following simulation-based training (22).

It is important to emphasize that the improvements reported in this study correspond exclusively to perceived competence. As the evaluation was limited to Level 1 of the Kirkpatrick model, no objective measures of skill acquisition, behavioral transfer, or patient outcomes were assessed. Therefore, the findings reflect participants’ reactions and self-perceived learning rather than demonstrable competency gains.

When analyzed by professional profile, no statistically significant differences were found between physicians and nurses, suggesting a very positive perception of the usefulness of clinical simulation within these clinical roles. Although comparative literature exists in undergraduate students (23), studies directly comparing satisfaction among practicing medical and nursing professionals remain scarce (24). In this regard, recent systematic reviews and meta-analyses confirm that interdisciplinary simulation improves interprofessional knowledge across different healthcare professional groups, though differences between profiles are observed depending on prior curricular exposure to simulation (25).

More refined analysis revealed differences when comparing the nursing group with the heterogeneous group comprising nursing assistants and other healthcare professionals. These profiles appear to perceive the impact of simulation-based training differently, which may be attributable to lower curricular exposure to simulation methodologies. To date, comparative evidence between these professional groups is limited, despite the need for their involvement to enhance teamwork, coordination, and consequently reduce errors within multidisciplinary teams.

When interpreting these differences, their magnitude should be considered alongside statistical significance. Although several comparisons reached statistical significance, most were associated with small effect sizes. Given the relatively large sample size (*n* = 542), the study had sufficient statistical power to detect small between-group differences. Therefore, while differences between certain professional categories were observed, their practical magnitude appears limited. Only the dimension related to the prioritization of clinical activities approached a moderate effect size. These findings suggest that perceptions of simulation-based training were generally similar across professional profiles, despite minor variations in specific domains.

The high overall ratings and perceived usefulness for clinical practice observed in our study are consistent with previous literature, which describes simulation as highly relevant for clinical decision-making and coordination (7, 8). These findings are also aligned with studies conducted in practicing nurses, which reported significant improvements in knowledge, skills, self-efficacy, confidence, and satisfaction following simulated clinical experiences, confirming the utility of this methodology beyond undergraduate training (26).

A distinctive element and key strength of this work is its multidisciplinary approach, supported by a broad sample that simultaneously integrates attending physicians, residents, nurses, and nursing assistants, allowing for the identification of satisfaction patterns in a real healthcare team. This approach adds value compared to much of the recent literature, where studies with unidisciplinary samples predominate, frequently focused exclusively on nursing students (9, 11, 16, 17) or medical students (19). While such works limit the understanding of impact to the training scope of a single collective, the results of the present work support the utility of simulation as a transversal strategy favoring team cohesion, as evidenced by interprofessional simulation programs integrating physicians, nurses, and respiratory therapists in hospital settings, where team communication and patient safety outcomes were significantly improved (27), overcoming the limitations of previous unidisciplinary approaches.

On the other hand, the lack of association between age and global satisfaction constitutes another relevant result, indicating that acceptance of the methodology is high regardless of the professional’s age. This is supported by meta-analyses of interprofessional simulation programs suggesting that the efficacy of simulation-based education depends primarily on the quality of program design and the clarity of educational objectives, rather than on individual participant characteristics such as age or prior familiarity with technology (28).

Finally, the experience of the center where the study was conducted demonstrates that it is possible to obtain elevated satisfaction levels by prioritizing methodology and teaching quality, even without having a space specifically dedicated to simulation. This finding resonates with evidence from hospital settings showing that implementing accessible simulation strategies without a dedicated simulation center is feasible and yields high perceived utility among healthcare professionals (29). This finding is especially pertinent given the context of structural challenges faced by many European and Spanish university hospitals described in the literature, including Spanish multicenter studies documenting logistical, temporal, and resource-related barriers to simulation in hospital environments, and demonstrating that *in situ* low-cost simulation programs can achieve high usefulness ratings despite such constraints (30).

Taken together, the presented results reinforce the utility and acceptance of clinical simulation as a multidisciplinary training strategy and underscore the importance of its systematic integration into professional development plans in hospital environments, even in resource-constrained settings.

## Limitations

This study presents several limitations that should be considered when interpreting the results.

First, the cross-sectional design of this study prevents establishing causal relationships between participation in simulation activities and the perceived outcomes. Although high satisfaction levels were observed, the design does not allow us to confirm that simulation training directly caused improvements in professional competencies. Longitudinal studies or controlled designs would be necessary to determine causal effects and evaluate sustained impact over time.

Secondly, satisfaction was measured using a self-administered instrument, which may introduce social desirability bias or initial motivation toward the training activity. Furthermore, the evaluation corresponds to Level 1 of the Kirkpatrick model, focused on satisfaction and perception of learning; therefore, the results do not allow for inferring objective improvements in clinical competencies or patient outcomes.

The use of non-probability sampling constitutes a limitation of the present design, given that the lack of randomization prevents guaranteeing full sample representativeness and may have introduced selection bias. This circumstance affects internal validity and limits the precision of comparisons between groups; thus, the conclusions should be interpreted with caution. Additionally, the aggregation of nursing assistants and other professional profiles into a single category may have introduced heterogeneity, potentially obscuring intra-group differences and limiting the interpretability of comparisons with other professional categories. This grouped category included administrative staff, midwives, nursing residents, and other healthcare personnel with potentially different roles, responsibilities, and prior exposure to simulation-based training. Such heterogeneity may have diluted specific subgroup effects and should be taken into account when interpreting between-group comparisons. A detailed breakdown of this category is provided in the supplementary material to enhance transparency. Future studies should consider analyzing these profiles separately to improve analytical precision.

Finally, although the questionnaire used is a validated tool for clinical simulation, it does not include specific dimensions such as teamwork or operational performance, meaning these aspects cannot be interpreted from the obtained data.

## Conclusion

This study observed high acceptance of clinical simulation among professionals of different categories, with consistently high satisfaction across all evaluated dimensions. Its main contribution lies in the multidisciplinary approach—integrating attending physicians, residents, nursing staff, and nursing assistants—an approach that remains relatively underexplored and suggests the transversal utility of simulation in real healthcare teams.

The homogeneity observed between physicians and nurses reinforces the cohesion of the clinical decision-making team, whereas the differences identified in the group of nursing assistants and other healthcare professionals highlight the need to make educational objectives more visible and to adapt them in order to ensure the full integration of these often underrepresented profiles. Furthermore, the high ratings obtained despite the absence of a dedicated simulation center underscore the importance of pedagogical design over infrastructure.

These findings suggest that structured clinical simulation may represent a valuable training strategy in hospital settings, particularly from the perspective of participant satisfaction. However, given the cross-sectional design and the focus on Level 1 outcomes, further research using longitudinal or controlled methodologies is required to confirm these benefits and to objectively evaluate skill acquisition, transfer to clinical practice, and long-term healthcare impact.

## Data Availability

The original contributions presented in the study are included in the article/supplementary material, further inquiries can be directed to the corresponding author.

## References

[1] Elendu C Amaechi DC Okatta AU Amaechi EC Elendu TC Ezeh CP . The impact of simulation-based training in medical education: a review. Medicine (Baltimore). (2024) 103:e38813. doi: 10.1097/MD.0000000000038813, 38968472 PMC11224887

[2] Herrera-Aliaga E Estrada LD. Trends and innovations of simulation for twenty first century medical education. Front Public Health. (2022) 10:619769. doi: 10.3389/fpubh.2022.619769, 35309206 PMC8929194

[3] Saleem M Khan Z. Healthcare simulation: an effective way of learning in health care. Pak J Med Sci. (2023) 39:1185–90. doi: 10.12669/pjms.39.4.7145, 37492303 PMC10364267

[4] Motola I Devine LA Chung HS Sullivan JE Issenberg SB. Simulation in healthcare education: a best evidence practical guide. AMEE guide no. 82. Med Teach. (2013) 35:e1511–30. doi: 10.3109/0142159X.2013.818632, 23941678

[5] Cook DA Hatala R Brydges R Zendejas B Szostek JH Wang AT . Technology-enhanced simulation for health professions education: a systematic review and meta-analysis. JAMA. (2011) 306:978–88. doi: 10.1001/jama.2011.1234, 21900138

[6] Issenberg SB McGaghie WC Petrusa ER Lee Gordon D Scalese RJ. Features and uses of high-fidelity medical simulations that lead to effective learning: a BEME systematic review. Med Teach. (2005) 27:10–28. doi: 10.1080/01421590500046924, 16147767

[7] Goldshtein D Krensky C Doshi S Perelman VS. In situ simulation and its effects on patient outcomes: a systematic review. BMJ Simul Technol Enhanc Learn. (2020) 6:3–9. doi: 10.1136/bmjstel-2018-000387, 35514456 PMC8936935

[8] Khanduja PK Bould MD Naik VN Hladkowicz E Boet S. The role of simulation in continuing medical education for acute care physicians: a systematic review. Crit Care Med. (2015) 43:186–93. doi: 10.1097/CCM.0000000000000672, 25343571

[9] Alharbi HF Alsubaie A Gharawi R Ba Mazroo R Alajaleen S Alsultan M . The relationship between virtual simulation, critical thinking, and self-directed learning abilities of nursing students in Riyadh. Saudi Arabia PeerJ. (2024) 12:e18150. doi: 10.7717/peerj.18150 39399417 PMC11470764

[10] Sellmann T Alchab S Wetzchewald D Meyer J Rassaf T Thal SC . Simulation-based randomized trial of medical emergency cognitive aids. Scand J Trauma Resusc Emerg Med (2022); 30:45. doi:10.1186/s13049-022-01028-y 35820939 PMC9277856

[11] Tong LK Li YY Au ML Ng WI Wang SC Liu Y . The effects of simulation-based education on undergraduate nursing students’ competences: a multicenter randomized controlled trial. BMC Nurs (2024); 23:400. doi:10.1186/s12912-024-02069-7 38886708 PMC11181658

[12] Constantino CS Genuino RF Kilem NKP Perias GAS Ang GGT. A scoping review on the status of clinical simulation in healthcare education in the Philippines. Acta Med Philipp (2025); 59:9–22. doi:10.47895/amp.v59i6.11554 PMC12174649 40538909

[13] Ferrari A Maglio S Tamirat S Tesfaye M Wolde M Manenti F . Nursing and midwifery simulation training with a newly developed low-cost high-fidelity placenta simulator: a collaboration between Italy and Ethiopia. BMC Med Educ. (2024) 24:1191. doi: 10.1186/s12909-024-06152-0, 39438956 PMC11515666

[14] Cascio M Ingrassia PL Zaccaria G . Role of simulation in Italian emergency medicine training programs: data from a national survey. Research Square[Preprint]. (2023). doi: 10.21203/rs.3.rs-2851075/v1

[15] Cánovas Zaldúa Y Martín Martín S Serraboguña Bret J Hermosilla Perez E Redon EC Rodoreda Noguerola S. Simulated clinical stations in quality and patient safety in a primary care setting. Healthcare (Basel) (2025); 13:1501. doi:10.3390/healthcare13131501 40648526 PMC12248645

[16] Alconero-Camarero AR Sarabia Cobo CM González-Gómez S Ibáñez-Rementería I Alvarez-García MP. Descriptive study of the satisfaction of nursing degree students in high-fidelity clinical simulation practices. Enferm Clin (Engl Ed). (2020); 30:404–410. English, Spanish. doi:10.1016/j.enfcli.2019.07.007 31443936

[17] Jiménez-Álvarez JA Guerra-Martín MD Borrallo-Riego Á. Nursing students’ satisfaction with clinical simulation: a cross-sectional observational study. Nurs Rep. (2024) 14:3178–90. doi: 10.3390/nursrep14040231, 39585122 PMC11587440

[18] Aebersold M. Simulation-based learning: no longer a novelty in undergraduate education. OJIN. (2018) 23. doi: 10.3912/OJIN.Vol23No02PPT39

[19] Padilla MJ González J Sarmiento F Tripoloni D Cohen Arazi L. Simulación clínica: Validación de encuesta de calidad y satisfacción en un grupo de estudiantes de Medicina. Revista Española De Educación Médica. (2023) 5. doi: 10.6018/edumed.591511

[20] Ingrassia PL Barelli A Benedetti E Bressan S Carenzo L D’Agostino F . A National Position Paper for the strategic development of health care simulation in Italy. J Patient Saf. (2026) 22:78–85. doi: 10.1097/PTS.0000000000001393, 40654313

[21] Yeh SL Lin CT Wang LH Lin CC Ma CT Han CY. The outcomes of an interprofessional simulation program for new graduate nurses. Int J Environ Res Public Health. (2022) 19:13839. doi: 10.3390/ijerph192113839, 36360719 PMC9653773

[22] Alrashidi N Pasay a Alrashedi MS . Effects of simulation in improving the self-confidence of student nurses in clinical practice: a systematic review. BMC Med Educ. (2023) 23:815. doi: 10.1186/s12909-023-04793-1 37904153 PMC10614341

[23] Yu J Lee W Kim M Choi S Lee S Kim S . Effectiveness of simulation-based interprofessional education for medical and nursing students in South Korea: a pre-post survey. BMC Med Educ. (2020) 20:476. doi: 10.1186/s12909-020-02395-9, 33243233 PMC7691096

[24] Alrashidi N Pasay An E Alrashedi MS Alqarni AS Gonzales F Bassuni EM . Effects of simulation in improving the selfconfidence of student nurses in clinical practice: a systematic review. BMC Med Educ (2023); 23:815. doi:10.1186/s12909-023-04793-1 37904153 PMC10614341

[25] Saragih ID Suarilah I Hsiao CT Fann WC Lee BO. Interdisciplinary simulation-based teaching and learning for healthcare professionals: a systematic review and meta-analysis of randomized controlled trials. Nurse Educ Pract. (2024) 76:103920. doi: 10.1016/j.nepr.2024.103920, 38382335

[26] Guerrero JG Attallah DM Gomma NH Ali SA. Improvements in practising nurses' knowledge, skills, self-efficacy, confidence, and satisfaction after a simulated clinical experience. BMC Nurs. (2024) 23:66. doi: 10.1186/s12912-024-01727-0, 38267925 PMC10807190

[27] Sung TC Hsu HC. Improving critical care teamwork: simulation-based Interprofessional training for enhanced communication and safety. J Multidiscip Healthc. (2025) 18:355–67. doi: 10.2147/JMDH.S500890, 39872869 PMC11769723

[28] Sezgin MG Bektas H. Effectiveness of interprofessional simulation-based education programs to improve teamwork and communication for students in the healthcare profession: a systematic review and meta-analysis of randomized controlled trials. Nurse Educ Today. (2023) 120:105619. doi: 10.1016/j.nedt.2022.105619, 36343420

[29] Trawber RAH Sweetman GM Proctor LR. Improving simulation accessibility in a hospital setting: implementing a simulation consultation service. Simul Healthc. (2021) 16:261–7. doi: 10.1097/SIH.0000000000000497, 32890318

[30] Butragueño Laiseca L Zanin A López-Herce Cid J Mencía BS. Simulation during COVID-19 pandemic in the Spanish pediatric intensive care units: new challenges in medical education. An Pediatr (Engl Ed). (2021) 95:373–5. doi: 10.1016/j.anpede.2021.06.007, 34688583 PMC8519807

